# Reducing Respiratory Health Risks to Horses and Workers: A Comparison of Two Stall Bedding Materials

**DOI:** 10.3390/ani5040394

**Published:** 2015-10-08

**Authors:** Markku Saastamoinen, Susanna Särkijärvi, Seppo Hyyppä

**Affiliations:** 1 Natural Resources Institute Finland (Luke), Opistontie 10 a 1, 32100 Ypäjä, Finland; E-Mail: susanna.sarkijarvi@luke.fi; 2 Ypäjä Equine College, Opistontie 9, 32100 Ypäjä, Finland; E-Mail: Seppo.hyyppa@hevosopisto.fi

**Keywords:** bedding material, respiratory health, peat, wood shaving

## Abstract

**Simple Summary:**

In this study, the effect of wood shavings and peat was examined on stable air quality and health of horses and stable workers. The ammonia level in the boxes in which peat was used as bedding was non-existent or very low. The respiratory symptoms in horses increased regardless of the bedding material at the beginning of the study. The health status of the horses on peat bedding returned to the initial level in the end of the trial but horses in stalls bedded with wood shavings continued to be symptomatic. The hooves of the horses in stalls with peat bedding had a better moisture content. The results suggest that peat is a better bedding material for horses and people working or visiting horse stables than wood shavings.

**Abstract:**

Stable air quality and the choice of bedding material are an important health issue both in horses and people working or visiting horse stables. Risks of impaired respiratory health are those that can especially be avoided by improving air quality in the stable. The choice of bedding material is particularly important in cold climate conditions; where horses are kept most of the day and year indoors throughout their life. This study examined the effect of two bedding materials; wood shavings and peat; on stable air quality and health of horses. Ammonia and dust levels were also measured to assess conditions in the stable. Ammonia was not detected or was at very low levels (<0.25 ppm) in the boxes in which peat was used as bedding; but its concentration was clearly higher (1.5–7.0 ppm) in stalls with wood shavings as bedding. Personal measurements of workers revealed quite high ammonia exposure (5.9 ppm_8h_) in the boxes in which wood shavings were used; but no exposure was observed in stalls bedded with peat. The respiratory symptoms in horses increased regardless of the bedding material at the beginning of the study. The health status of the horses in the peat bedding group returned to the initial level in the end of the trial but horses bedded with wood shavings continued to be symptomatic. The hooves of the horses with peat bedding had a better moisture content than those of the horses bedded with wood shavings. The results suggest that peat is a better bedding material for horses than wood shavings regarding the health of both horses and stable workers.

## 1. Introduction

The stalls of horses are bedded to absorb urine, moisture, and gases and to increase the comfort, health, and well-being of the horses. In addition, a large number of people are engaged in the horse industry as trainers, riders, stable workers, farriers, and other roles working or visiting stables for many hours daily, and being subjected to the influences of the stable environment. Most of them are young people, for example riding school pupils.

Horses in northern climatic conditions (e.g., in the Nordic countries and Canada) are exercised outdoors usually for 1 to 2 h and spend, consequently, the major part of the day (often up to 23 h) indoors. Because of this, stable air quality is of considerable importance. Furthermore, training and racing in cold weather conditions expose the vulnerable respiratory system to health problems, increasing inflammatory cells in the lungs [1]. As a consequence, respiratory disorders are common problems, and respiratory allergy is commonly diagnosed as a condition affecting the equine lung. When the condition becomes protracted it is referred to as chronic obstructive pulmonary disease (COPD) or heaves (or RAO, recurrent airway obstruction), an animal model of asthma. Anecdotal reports suggest that the condition is rare in climates where animals are outside all year around but is common in climates where horses are stabled indoors [2]. Clinical signs in horses with this chronic lung disease include poor athletic performance, chronic couching, purulent nasal discharge, and ultimately difficulties in breathing [2,3].

People working in and visiting horse stables may also be exposed to the effects of the stable air. Causes of chronic airway disease both in horses and humans usually involve exposure to excessive concentrations of airborne dust, molds, viruses, bacteria, spores, aeroallergens, and endotoxins which mostly originate from bedding and feed [4,5,6,7]. Furthermore, the inhalation of gaseous irritants such as ammonia may initiate airway obstruction and exacerbate or prolong the clinical signs of COPD in affected horses [2] as well as humans [3].

The effect of bedding material on the quality of stable air, both on stable dust and the ammonia concentration, is significant [8]. The various forms of bedding in a stable and even the differences in beddings between boxes within a stable [8] influence the stable dust and gas loads, and consequently the risk to airway health of both horses and humans.

Currently, several materials are available for the bedding of boxes in horse stables. The most common bedding materials are wood shavings, saw dust, straw, and peat. Many other materials are also used as bedding, including processed (pelleted) wooden materials and (pelleted, chopped) straw from different plants. In addition, shredded or cut paper and some plant materials (by-products), as well as woodchips are used. Each of these has individual properties, including advantages and disadvantages [9]. Stall mats are also available, but they are usually used together with bedding because of the binding of urine. It has also been reported that horses prefer bedding material for lying down as compared with areas without it [10].

Factors considered when selecting bedding are its availability, cost, cleanness (free from dust and foreign material) and its effect on stable air quality. The bedding material should also be easy and light to handle, to avoid excessive physical exertion in stable workers. From the point of view of horses’ health and well-being, good bedding creates a layer of insulation between the horse and cold floor, pads the hard surface, prevents bruised knees, elbows, hocks and hips, and keeps the horse clean. The bedding material also affects the behavior of horses [11,12,13], for example the incidence of stereotypic behaviour. In addition, good bedding material has a better potential to be re-used e.g., in farming and horticulture [14,15].

The objective of this study was to examine the effects of two different bedding materials, wood shavings and peat, on the health of horses. This issue was evaluated on the basis of respiratory and overall health and quality of hooves, and by measuring stable air quality.

## 2. Materials and Methods

The experiment was conducted in the facilities of MTT Agrifood Research Finland (MTT, currently Natural Resources Institute Finland Luke) in the south western part of Finland (latitude 60°) under autumn/early winter (October to December) climatic conditions. The duration of the experiment was 84 days. Twelve Finnhorse brood mares (four of which were pregnant) aged between 5 and 17 years were housed in box stables in individual stalls (3 m × 3 m), divided into separate sections of the stable according to the bedding material (peat; wood shavings). The stable sections were of the same size and had an identical mechanical ventilation system. The horses were held on pasture from the beginning of June to the middle of September.

The two bedding materials were selected because they are the most common materials used for bedding in Finland. They both have a low content of harmful components when manufactured, selected and stored properly. Peat is favoured as a bedding material because of its good properties in soil improvement and good composting ability, as well as its superior capacity to bind ammonia and fluid [13,16,17] compared to other materials. Both bedding materials were manufactured for use as beddings in horse stalls; peat by Vapo Ltd. (Jyväskylä, Finland) and wood shavings by Joutsenon purupaali Ltd. (Joutseno, Finland).

The horses were exercised daily in paddocks in groups for four hours, and for one hour by riding or driving during the course of the experiment. The stalls were manually cleaned by the same person between 8 and 12 am when the horses were in outdoor paddocks. All feces and wet material were removed and new bedding material was added. The depth of the bedding was about 10 cm. All removed and added bedding materials were measured by their volume.

The horses were individually fed according to their needs three times per day (morning, noon, evening) with silage/haylage (DM 26.6%–6.9%) and pelleted compounded feed (DM 88%) (Suomen Rehu Ltd., Turku, Finland) to minimize the release of airborne particles from the feeds. The diet was balanced for protein (nitrogen) intake to avoid nitrogen lost in urine and, thus, to minimize the ammonia in the stable. The forage was produced by MTT and its fermentation and hygienic quality fulfilled the criteria of good quality haylage and silage [18]. The forage was placed on the floor.

Outdoor temperatures and weather conditions were recorded daily at 8:00 am. The average outdoor temperatures in October, November and December, respectively, were −1.7 °C (−10 to 2 °C), −3.8 °C (−14 to 3 °C) and −6.6 °C (−20 to 0 °C). According to the statistics of the Finnish Meteorological Institute the temperatures in December were quite normal, but in November the daily temperatures were highly variable, and in October the temperatures were exceptionally low.

The stable temperatures and humidity, as well as ammonia and carbon dioxide levels and amount of dust, were measured daily in both stable sections. Methane (day 0) and hydrogen sulphide (days 0 and 42) contents were measured, but because of undetectable values the measuring was not continued. Gases concentrations were measured at a height of 120 cm from the ground using an Accuro gas detection pump which draws air through sampling tubes (Dräger Safety AG, Lübeck, Germany). The measurements were carried out at 6:00 am in three boxes of each stable section; from the middle of the box at the level of the muzzle of the horse. Dust was continuously collected into dust cases that were fitted in empty boxes in both stable sections at the level of 40 cm from the ground.

Exposure of the stable workers to ammonia was evaluated with personal measurements using sampling tubes attached to the lapel of the person (Dräger Safety AG, Germany) in the middle and at the end of the experiment during the cleaning of the stalls. The measurement result was converted to correspond to an exposure period of eight hours (HTP_8h_) [19]. HTP value is the concentration that is harmful to people.

A respiratory endoscopic examination was performed three times during the study (days 0, 42 and 84), including examination of the ethmoidal region, pharyngeal openings of guttural pouches, soft palate, larynx, and trachea (symptoms = 1; no symptoms = 0). Tracheobronchial aspirates were drawn during the endoscopy and cytological and bacteriological (neutrophil cells) evaluation was carried out. The classification of the neutrophil cells in bronchoalveolar smear samples was as follows: none or some single cells (−); single cells and few small pool of cells (+); several large pools of cells (++); abundant pools of cells (+++); and an extreme abundance of cells (++++).

Blood analyses, fecal analyses and hoof quality evaluation were used as measures of health and well-being of the horses. These samples were taken with the same interval as the endoscopic examination. In addition, rectal body temperature was measured and, heart rate (with stethoscope) and respiration rate via auscultation were recorded by a veterinarian researcher. Blood samples were collected from the jugular vein, and the blood analysis consisted of hemoglobin, haematocrit, serum urea, iron, protein, and differential cell count. Bacteriology, parasites, and the pH of faeces were determined. The quality of hooves was assessed from the dry matter content of hoof horn. The hoof horn samples were collected from the hooves of front legs when the horses were in shoeing. All samples were analyzed in the clinical laboratory of MTT.

The experimental design was a randomized block design with repeated measurements. After the first endoscopy, the horses were formed into pairs based on their symptomatic similarity. The two horses of each pair were then randomly allotted to different bedding material groups (peat bedding or wood shaving bedding). The procedure was repeated until all horses were divided in the two groups. The information from the first endoscopy was excluded from the data because it was included in the animal pair-variable in the model. The data (samples from horses) were analyzed using the MIXED procedure of the SAS system with the following statistical model: *Y_ijk_ =* µ *+ p_i_ + b_j_ +* (*p × b*)*_ij_ + t_k_ +* (*p × t*)*_jk_ +* (*b × t*)*_jk_ + e_ijk_*, where *Y_ijk_* is the observation, µ is the overall mean, *p_i_* is the random effect of *i^th^* animal pair (*i* = 1 … 6), *b_j_* is the fixed effect of *j^th^* bedding material (*j* = 1 … 2), *t_k_* is the fixed effect of the time period (*k* = 2 or 3), and *e_ijk_* is the normally distributed error with a mean of 0 and variance δ^2^. Terms (*p × b*)*_ij_*, (*p × t*)*_jk_* and (*b × t*)*_jk_* are compound effects of factors. The best fitting covariance structure for repeated measurements was selected on the basis of the Akaike information criterion. The differences were tested with Tukey’s test. Categorical variables (neutrophil cells in tracheal mucus) and 0/1-variables were not tested statistically, but were presented descriptively, because of the small number of observations and their subjective scoring making them less informative.

In animal handling and sample collection, the European Union recommendation directives (1999/575/EU) and national animal welfare and ethical legislation set by the Ministry of Agriculture and Forestry of Finland were followed carefully. The experimental procedures were evaluated and approved by The Animal Care Committee of MTT before the study was started. The endoscopic examination was carried out and all samples from the horses were collected by a veterinarian researcher.

## 3. Results and Discussion

### 3.1. Air Quality

The average temperatures in the stable sections (peat bedding *vs.* wood shaving bedding) in October, November and December, respectively, were 9.2 *vs.* 10.3 °C, 9.4 *vs.* 9.4 °C, and 8.0 *vs.* 8.0 °C. These temperatures are within the target indoor temperature range (8–12 °C) in horse stables in Finland [20]. The average moisture content of the stable air (peat *vs.* wood shavings) was 54.3% *vs.* 54.6%, 56.0% *vs.* 57.6% and 53.0% *vs.* 58.6% in the corresponding months. During the lowest outdoor temperatures the moisture contents were naturally at the lowest levels (38% to 44%). The moisture of the stable air originates from horses’ respiration, urine, feces and drinking and washing water. Excessively high temperatures and moisture may increase the release of ammonia from the bedding [21].

The bedding material numerically influenced the ammonia content of the stable air (Table 1). Measurements we made early in the morning before any other activity in the stables. Thus, the ammonia concentrations represent the situation at its worst after the night. The ammonia level in the middle of the boxes in which peat was used as bedding was non-existent or very low (<0.25 ppm). However, the ammonia concentration in the stalls with wood shavings as bedding was numerically (6–8 times) higher (1.5–7.0 ppm) and at the highest close to levels (10 ppm) considered harmful [22]. The ammonia levels observed in the present study were lower than recently reported gas levels in the morning in stables with bedding consisting of pine wood shaving [23], but under warmer conditions (summer, in North Dakota, US). Ammonia in the stable originates from urine. The urinary production depends the diet (N-intake) and water intake. Both urinary production and N-losses increase with increasing N-intake [24]. In the present study the diet was individually balanced for protein (N) intake, and excretion of N was not obviously very high. There were no differences in ammonia concentrations in the stalls of horses on peat bedding (0–0.25 ppm) because of the superior ammonia absorption capacity of peat. Concerning the horses on wood shavings bedding the ammonia content varied between the horses (stalls) and measuring dates from 1.5 to 7.0 ppm.

**Table 1:** Gas concentrations in the stable air.

	Wood Shaving Bedding	Peat Bedding
Day 0		
Ammonia	0.5 ppm	0 ppm
Carbon dioxide	650 ppm	500 ppm
Hydrogen sulphide	0 ppm	0 ppm
Methane	0 ppm	0 ppm
Day 42		
Ammonia	1.5–7.0 ppm	0–0.25 ppm
Carbon dioxide	500 ppm	700 ppm
Hydrogen sulphide	0 ppm	0 ppm
Day 84		
Ammonia	4.0–7.0 ppm	0–0.25 ppm
Carbon dioxide	700 ppm	600 ppm

The amount of dust collected was small for both bedding types, and no major differences were observed in dust measurements between the bedding materials. Both bedding materials were specially manufactured for use in horse stalls. The carbon dioxide values were lower than the upper acceptable limit values for horses (3000 ppm) and for humans (1000 ppm) [3]. Carbon dioxide levels were similar in both stable sections.

The bedding materials also affected the environment of the people working in the stables. Personal measurements of the ammonium exposure of the workers revealed was higher (5.9 ppm_8h_) in the boxes in which wood shavings were used. No exposure was observed in stalls bedded with peat. This is important to consider, because workers can spend a considerable amount of time each day in the stables. In this study it took about 13 min to clean one box. According to previous studies, feeding and handling of feed take about 5 to 7 min per horse per day [25,26], and cleaning the stalls (mucking out, replacement of bedding materials) takes approximately 10 min per horse daily if no machinery is used [25,26,27]. The upper limit of HTP_8h_ is, however, as high as 20 ppm [19].

Studies on peat as a bedding material are scarce. Airaksinen *et al.* [17] and Nikama *et al.* [15] have reported a superior ability of peat bedding to bind ammonia, which is based on its low pH value. The pH value of the peat for bedding (Vapo Ltd.) used in the present study was 4.0. The pH value of wood shavings used here was not available, but according to a study the pH of wood shavings is higher (pH 5.5) than that of peat [28]. Peat tended to create a better stable environment than pelleted sawdust due to higher absorption of ammonia and lower levels volatile organic compounds [3], but no differences between the bedding materials were observed regarding the amount of dust. However, depending on its origin, peat has been shown to vary widely in dustiness and hygienic quality [17].

According to several other studies, the type of the bedding material has a considerable effect on stable dust, ammonia, bacteria, and endotoxin concentrations in horse stalls [4,7,23,29,30,31]. Fleming *et al.* [7] observed that the gaseous ammonia concentration was lowest when straw pellets were used. The order based on ammonia concentrations among the studied bedding materials in their study was straw pellets, linen, hemp, wood shavings, paper cuttings, and wheat straw. In a study by Garlipp *et al.* [31] ammonia emissions from wood shavings were considerably lower than from straw. In some studies mucking out and handling of bedding materials influenced the dust and gas (ammonia) emissions in the stable [7,30,32]. In the present study, the ammonia content of the stable air was higher when wood shavings were used, and during mucking exposure of ammonia occurred only in stalls bedded with wood shavings, resulting from the superior ammonia binding capacity of peat.

Pelleting of the bedding material reduces the generation of airborne particles by the bedding material [33]. Fleming *et al.* [30] found the lowest particle generation with straw pellets. In their two studies [7,30] they concluded that straw pellets may promote an improvement in the stable climate in relation to airborne particle formation, ammonia binding and ammonia transformation. Pelleted newspaper also appears to have a good potential as a bedding material for horses [33].

In one study [34] the researchers observed that the generation of airborne particles in straw, wood shavings, flax, and hemp can be reduced with a separation technology. They also found that the generation of particles increased during the storage of the bedding.

Proper ventilation is important to remove moisture, gases, and dust and other particles from the stable. However, in many cases the ventilation of stables does not provide adequate exchange of fresh air. Thus, the quality and properties of the bedding material are of considerable importance.

### 3.2. Horse Health

The first endoscopic examination at the beginning of the experiment revealed that 4 of the 12 horses had respiratory symptoms (+…+++). Thus, moving the horses from pasture to indoor housing in the middle of September (two weeks before beginning of the study) appeared to expose the horses to respiratory disease because of the air quality of the stable. In a Swedish study the highest dust measurements were observed in winter when the stable doors were closed [3]. Slightly increased airborne bacteria levels were also observed in stables in September compared to other seasons in that study. In the present study the frequency of respiratory symptoms increased in both groups during the first half of the study period, but then decreased in the horses bedded with peat such that the number of horses with symptoms in this group was the same at the beginning and end of the experiment. In the horses bedded with wood shavings the number of symptomatic horses remained larger than at the beginning, being twice of that compared to the horses with peat bedding (Table 2). Thus, the peat bedding seemed to be a better bedding choice than wood shavings regarding the health of respiratory tract.

The number of neutrophil cells did not differ between the groups (data not shown). The tracheobronchial aspirates obtained during endoscopy contained either scarce or moderate numbers of neutrophils (peat bedding: −…++; wood shavings bedding: 0…+++). An elevated number of neutrophils or the detection of Curschman’s spirals is suggested to correlate with CODP symptoms [35]. One of the horses bedded with wood shavings had a high neutrophil percentage and also spirals in its sample at the second and third samplings, and was therefore diagnosed as a CODP horse at that time.

**Table 2:** Symptomatic horses based on endoscopy examination.

Wood Shaving Bedding Horse	Day 0	Day 42	Day 84
1	1	1	1
2	0	0	0
3	0	0	1
4	1	1	1
5	0	1	0
6	0	1	1
Horses with symptoms	2	4	4
Total symptomatic during the experiment			10
Peat bedding Horse	Day 0	Day 42	Day 84
7	0	1	0
8	1	1	1
9	0	1	0
10	0	1	1
11	0	0	0
12	1	0	0
Horses with symptoms	2	4	2
Total symptomatic during the experiment			8

There were no statistically significant differences in blood parameters of the horses between the peat bedding and wood shaving bedding groups (data not shown). The parameters in all horses were within the range of reference values. The bedding did not affect the respiration or heart rate of the horses (data not shown). The microflora of feces was also unaffected by the bedding, which is in agreement with Tanner *et al.* [4] and Hübinette [36]. The pH value was somewhat lower (*p* = 0.01) in the horses with peat bedding (6.78) than in those bedded with wood shavings (7.08) at the end of the experiment (day 84), and the colour of their feces was darker, which was obviously a result of observed eating peat in small amounts. Hübinette [36] found no effect of bedding material (wood shavings or peat) on the faecal pH.

The moisture content of the hoof horn at the end of the study was higher (*p* < 0.05) in the horses bedded with peat (32.6%) compared to the horses with wood shavings as bedding (30.5%). In the middle of the study the difference was not statistically significant (peat 34.2%, wood shavings 33.2%). Dryness of the hooves can cause problems when the natural elasticity and toughness is lost [37]. The weakening of the hoof mechanism can lead to hoof cracks and impose an additional strain on the legs. Tanner *et al.* [38] found that the hooves were dryer and more caked when phone book paper was used as a bedding material than in horses bedded on sawdust.

The results supported by literature [17,30] suggest that bedding materials have the potential to affect stable air conditions and animal health and welfare. However, in some studies, no differences have been observed. For example Tanner *et al.* [38] found no difference in the respiratory health of horses when bedded with either sawdust or (shredded and milled) phonebook paper. However, the choice of bedding material is especially important in cold climatic conditions, which forces horses to be kept indoors for a large part of the year.

Although there appears to be clear differences in the properties and influences between various bedding materials and types, the quality and origin of a particular bedding is important regarding the airborne dust concentrations originating from the bedding material [17,30,39], and stable owners and managers should thus also pay particular attention to this issue when selecting bedding materials.

Horses appear to have individual preferences for bedding material, and no significant overall preference for example for either wood shavings or straw was observed [12]. Werhan *et al.* [13] also found individual differences, but the horses generally preferred straw. The authors concluded that on the basis of the longest time being occupied, straw seems to support the welfare of the horse better than wood shavings or straw pellets.

### 3.3. Consumption of the Bedding Materials

The consumption of bedding materials differed considerably. The consumption of peat was 59% of that of wood shavings (by volume), obviously due to its superior ability to bind liquids. This affects the cost of bedding as well as the need for storage for both bedding material and manure, thus influencing the construction costs of the facilities.

### 3.4. General Discussion

An issue of increasing importance is the influence of manure on environment, which means that the amount of manure produced should be minimal and that it should be easily used as a fertilizer or in soil improvement [14,15], or even as a source of energy, for example in methane production [40]. Rapid composting and a good ability to bind and transfer nitrogen are important properties of peat bedding requested by farmers and other users of manure. Poeplau *et al.* [41] reported positive trends in organic carbon storage in Swedish agricultural soils due to increased horse industry and horse manure use in agriculture during the past two decades. It is also important that the bedding material can be easily handled in the stable, which is influenced, for example, by how it is packed, or how much it is consumed daily.

Peat is a good alternative for bedding material in those countries where it is produced for agricultural/horticultural or energy use, for example in the Nordic and Baltic countries, Russia, Poland *etc.* However, it is important to consider that agricultural peat soils should be managed sustainably and that cultural and socio-economical aspects of peatlands are taken into account [42]. In many other countries, such as in The Netherlands and Germany, the percentage of remaining pristine mires is small [43].

The battery of methods used to monitor indoor air quality and animal health in the stable was limited in the present study. In addition, outdoor exercise of the horses makes this issue complicated to investigate, regardless of the methods applied. Horses also individually differ in their sensitivity to exposure to environmental factors, *i.e.*, their genetic predisposition for example, to RAO [44]. On the other hand, many studies regarding airway health have been field studies without a controlled or standardised environment, e.g., examining bedding and feeding practices and outdoor exercise. The findings of the present study suggest that further research with a large sample size would be warranted in order to gain a better understanding on the effect of bedding materials on stable air quality and health and wellbeing of horses.

## 4. Conclusions

The results suggest that choice of bedding material is of large importance regarding stable air quality, at least in terms of the ammonia level. Both horses and people working in stables are exposed to ammonia if the ability of the bedding material to bind gases and fluids is poor. This may predispose both horses and humans to airway diseases. Based on the results, peat is superior to wood shavings regarding the ability to bind ammonia and reduce ammonia concentration and the risk of ammonium exposure of horses and stable workers. It seems also that horses on peat bedding may have better airway health. The moisture content of the hooves of the horses on peat bedding was higher compared to those bedded on wood shavings. The findings suggest that further research with a larger sample size is warranted.
